# Improved DNA extraction technique from clot for the diagnosis of Chagas disease

**DOI:** 10.1371/journal.pntd.0007024

**Published:** 2019-01-11

**Authors:** Holger Mayta, Yomara K. Romero, Alejandra Pando, Manuela Verastegui, Freddy Tinajeros, Ricardo Bozo, Josephine Henderson-Frost, Rony Colanzi, Jorge Flores, Richard Lerner, Caryn Bern, Robert H. Gilman, for the Chagas Working Group in Perú and Bolivia

**Affiliations:** 1 Infectious Diseases Research Laboratory, Department of Cellular and Molecular Sciences, School of Science and Philosophy, Universidad Peruana Cayetano Heredia, Lima, Peru; 2 Department of International Health, Johns Hopkins University Bloomberg School of Public Health, Baltimore, Maryland, United States of America; 3 A.B Prisma, Lima, Perú; 4 Hospital Municipal Camiri, Camiri, Plurinational State of Bolivia; 5 Massachusetts General Hospital, Boston, Massachusetts, United States of America; 6 Hospital Universitario Japones, Santa Cruz de la Sierra, Plurinational State of Bolivia; 7 Hospital San Juan de Dios, Santa Cruz de la Sierra, Plurinational State of Bolivia; 8 Pan American Zoonotic Research and Prevention, Framingham, Massachusetts, United States of America; 9 Department of Epidemiology and Biostatistics, University of California—San Francisco, San Francisco, California, United States of America; Center for Biologics Evaluation and Research, Food and Drug Administration, UNITED STATES

## Abstract

**Background:**

The detection of *Trypanosoma cruzi* genetic material in clinical samples is considered an important diagnostic tool for Chagas disease. We have previously demonstrated that PCR using clot samples yields greater sensitivity than either buffy coat or whole blood samples. However, phenol-chloroform DNA extraction from clot samples is difficult and toxic. The objective of the present study was to improve and develop a more sensitive method to recover parasite DNA from clot samples for the diagnosis of Chagas disease.

**Methodology/Principal findings:**

A total of 265 match pair samples of whole blood–guanidine (GEB) and clot samples were analyzed; 150 were from Chagas seropositive subjects. DNA was extracted from both whole blood-guanidine samples, using a previously standardized methodology, and from clot samples, using a newly developed methodology based on a combination of the FastPrep technique and the standard method for GEB extraction. A qPCR targeting the nuclear satellite sequences was used to compare the sample source and the extraction method. Of the 150 samples from Chagas positive individuals by serology, 47 samples tested positive by qPCR with DNA extracted by both GEB and clot, but an additional 13 samples tested positive only in DNA extracted from clot. No serology-negative samples resulted positive when tested by qPCR.

**Conclusions:**

The new methodology for DNA extraction from clot samples improves the molecular diagnosis of Chagas disease.

## Author summary

Detection of nucleic acid has become an important tool for the diagnosis of Chagas disease. Whole blood samples are usually the source of DNA and qPCR the preferred technique to demonstrate the presence of *T*. *cruzi* DNA. Although DNA extracted from clot samples has shown higher sensitivity than from whole blood, DNA extraction is performed using phenol-chloroform, which has biohazard issues. We theorize that a clot traps parasites, making it a better source of DNA for Chagas diagnosis using PCR. The present study describes a new DNA extraction methodology from clot samples which avoids the use of phenol-chloroform. The new methodology was compared to the internationally standardized diagnostic method, which is based on extraction of DNA from whole blood preserved with guanidine EDTA and a commercial kit.

## Introduction

Chagas disease, caused by the protozoan parasite *Trypanosoma cruzi*, is endemic in many parts of the Americas [1,2], where 6 to 7 million people are infected [3]. In the acute phase of the infection, serology by IgG may still be negative but positive for parasite by microscopic examination or by culture in specialized medium [1,4]. Infected individuals then enter a chronic phase where parasites are rarely seen in the blood and diagnosis relies on the use of serological tests. About 20–30% of infected individuals will develop cardiomyopathy during the subsequent chronic phase, the most important consequence of Chagas disease [1,2,5].

The Polymerase Chain Reaction (PCR) has become an important and sensitive diagnostic tool. The test is highly sensitive for diagnosis of acute and congenital Chagas disease [1]. Serial monitoring by quantitative PCR provides the earliest indication of Chagas disease reactivation [6]. PCR sensitivity during the chronic phase is highly variable depending on several factors such as volume and processing of specimens, characteristics of the analyzed population, primers and PCR methods. Negative PCR results do not prove that infection is absent. Systematic monitoring by means of PCR of serial blood specimens has been suggested to improve PCR diagnosis [1,7,8].

Conventional PCR has several drawbacks, especially cross-contamination. For this reason, quantitative PCR (qPCR) has now been more widely used and accepted. For Chagas disease, the qPCR is now increasingly used as a research tool for monitoring the disease [4] and also as a primary outcome in clinical trials for new drug candidates [9–11].

To achieve a high sensitivity of PCR analysis, efficient nucleic acid purification is required. Whole blood samples are primarily the main source of DNA for diagnosis. It has been recommended that when blood samples are drawn they should be immediately mixed with one volume of 6M guanidine hydrochloride– 0.2 M EDTA, pH 8.00 (GE) to stabilize DNA for shipping [12,13]. However, shipping and handling of guanidine is troublesome and the logistics for specimen collection with GE are complex. Recently we have demonstrated, by conventional PCR, that clot samples are superior as a source of DNA for Chagas diagnosis [14]. However, DNA extraction was performed using the phenol–chloroform method, which has several disadvantages: the extraction process is complex, the clot needs to be washed, the reagents are toxic, and the quantification cycle (Cq) values of qPCR assays are variable when the assay is repeated.

The objective of the present study was to improve and develop a more sensitive method to recover parasite DNA from clot samples for the diagnosis of Chagas disease. Clot was initially disrupted using the FastPrep technique and then DNA was extracted using the recommended methodology of the Roche extraction kit. DNA extracted from clot proved to have a higher sensitivity and presented a lower Cq value compared to DNA extracted from whole blood-GE (GEB) samples.

## Materials and methods

### Clinical samples

A total of 265 pairs of samples (one GEB and a clot sample) were obtained from archived specimens in our biorepository. The samples were obtained from two sample sets: Archived samples from women presenting for delivery at the Hospital Municipal Camiri in Camiri, Bolivia (n = 100) [15]; and samples collected at the Hospital Municipal San Juan de Dios, Santa Cruz, Bolivia (n = 165) [16,17].

### Ethics statement

The study protocol at the Hospital Municipal Camiri was approved by Institutional Review Board of Johns Hopkins Bloomberg School of Public Health, Asociacion Benefica PRISMA (Lima, Peru), and Universidad Catolica Boliviana (Santa Cruz, Bolivia).

The study protocol at the Hospital Municipal San Juan de Dios was approved by the Institutional Review Board of Johns Hopkins Bloomberg School of Public Health (Baltimore, MD, USA), Universidad Catolica Boliviana (Santa Cruz, Bolivia), and University of California San Francisco School of Medicine (San Francisco, CA, USA).

### Sample collection

Peripheral blood from individuals suspected of Chagas diseases was collected in tubes with EDTA and tubes without additives. The blood obtained without additives was allowed to sit for at least 30 min to let the clot to form (no more than 60 min) and then centrifuged at 1100 x g for 20 min [18]. After transferring the serum, the clot was frozen at –80°C and shipped with dry ice to the Infectious Research Laboratory at the Universidad Peruana Cayetano Heredia in Lima, Perú. Upon arrival, samples were immediately stored at -80°C until use. The blood obtained with EDTA was immediately mixed with one volume of guanidine hydrochloride 6M/EDTA 0.2M, pH = 8.0 (GE), shipped and stored at 4°C until use [19].

### DNA extraction

Blood samples mixed with Guanidine/EDTA (GEB) were processed using the High Pure PCR Template Preparation kit (Roche Diagnostics GmbH, Mannheim, Germany) as previously described [12,20]. Extraction was performed using 300 μl of GEB and 5 μl (40 ρg/μl) of an internal amplification control (IAC), were added to each sample at the beginning of the purification process to ensure the quality of the DNA purification process and the absence of PCR inhibitors.

Clot samples were initially homogenized using the FastPrep machine followed by extraction using the High Pure PCR Template Preparation kit. Briefly, 300 μl of clot, 300 μl of Guanidine/EDTA (GE), 40 μl of Proteinase K (20 mg/ml) and 5 μl of IAC (40 ρg/μl) were place in a Lysing Matrix E tube (MP Biomedicals, Santa Ana, CA). The mixture was FastPrep processed at 5,5 m/s for 30 sec and then centrifuged at 14000 x g for 2 min at room temperature. A total of 450 μl of supernatant was transferred to a new tube and 150 μl of binding buffer from the High Pure PCR template preparation kit (Roche Diagnostics) was added. The mixture was incubated at 70 °C for 10 min and the extraction process was performed using High Pure PCR Template Preparation kit (Roche Diagnostics).

Initially Lysing Matrix C, F, H and J were tested using spiked blood clot, only Lysing Matrix E and Lysing Matrix H gave comparable results as measured by the Cq values. However, Lysing Matrix E was chosen because the results were more reproducible.

### Spiked blood samples

The standard curves for parasitic load quantification were built using an isolate (BIO-6398) from Santa Cruz, Bolivia; identified as TcDTU V following the previously described typing methodology [21]. To build the curve 9 ml of whole blood extracted with EDTA from seronegative subjects was spiked with 1 ml of 10^6^ cultured epimastigote-stage parasites/ml suspended in PBS [22]. After properly mixing the sample the DNA was extracted as described for GEB clinical samples.

Similarly, to build a standard curve for clot samples, 9 ml of blood extracted without additives was spiked with 1 ml of 10^6^ parasites/ml suspended in PBS immediately after the blood was drawn and thoroughly mixed by inverting the tube for at least 20 times. The spiked sample was then allowed to sit for at least 30 min to let the clot to form (no more than 60 min). Clot was then recovered after centrifugation at 1100 x g for 20 min. DNA from clot was extracted as described for clinical clot samples.

### Internal amplification control

To assure adequate DNA purification, a recombinant plasmid (pZErO-2) containing the aquaporin gene of *Arabidopsis thaliana* as a heterologous extrinsic internal amplification control (IAC) was used [12,20,23]. The recombinant plasmid was provided by Dr. Alejandro Schijman’s laboratory (INGEBI-CONICET, Argentina).

### Other diagnostic assays

The diagnosis of Chagas disease in human samples was based on serological assays. ELISA was performed using Chagatek Wiener Recombinante v3.0 ELISA (Wiener laboratories, Rosario–Argentina). Indirect hemagglutination assay (IHA) was performed using the Chagas Polychaco kit (Lemos Laboratories, Buenos Aires–Argentina). Western blot analysis was performed using the TESA antigen harvested from *T*. *cruzi* Y strain growth in LLC-MK2 cells as previously described [24].

### Real time PCR

A duplex qPCR was performed targeting the satellite sequence of the nuclear genome of *T*. *cruzi* and the sequence of an internal amplification control [25]. The qPCR reaction was carried out as previously described using 5 μl of re-suspended DNA in a final volumne of 20 μl [12]. The Mastermix consisted of 1X FastStart Essential DNA Probes Master (Roche Diagnostics GmbH Corp., Mannheim, Germany), 0.75 μM of each primer Cruzi1 and Cruzi2, 0.1 μM of each primer IACFor and IACRev, and 0.5 μM of each Cruzi3 probe and IACtq probe. The cycling conditions were a first step of 10 min at 95 °C followed by 40 cycles at 95 °C for 15 sec and 58 °C for 1 minute.

### Data analysis

The qPCR for a sample was considered as valid when the Cq corresponding to the amplification of its IAC was lower than the 75^th^ percentile plus 1.5 the interquartile range of the median of each extraction batch (CqIAC<75^th^ percentile + 1.5*IQR).

The Cq values for the positive clinical sample were normalized respect to the efficiency of the DNA extraction procedure measured by the amplification of the IAC. The following formula was used: Cq_nor_ = Cq IAC_pos_ / CqIAC_neg_, where Cq_nor_ is the normalized Cq value for a given positive sample, Cq IAC_pos_ is the mean Cq IAC value for the positive controls, and the CqIAC_neg_ is the mean Cq IAC value for the negative controls included in the plate.

Statistical analysis was performed using Stata14 (Stata Corp, College Station, TX). Two-way tabulations of frequencies and means were performed. Results of the initial testing of the different lysing matrix were compared using the Kruskal Wallis test. Confidence intervals and McNemar’s test were used to compare the frequency of PCR results. Mean Cq values were compare using McNemar’s test. Agreement between qPCR results from clot and GEB samples was assessed using kappa statistic.

## Results

No differences on the Cq values were observed when qPCR for *T*. *cruzi* was performed on DNA extracted using lysing matrix C, H or J from clot samples spiked with 5 x 10^6^ parasites (*P = 0*.*13*, S1A Fig), however the use of lysing matrix C and J were discarded because of destruction of the lysing matrix components during FastPrep process. The Cq values on the qPCR for *T*. *cruzi* were lower on DNA extracted using lysing matrix E than F from clot samples spiked with 1 x 10^6^ parasites/ml (*P<0*.*001*, S1B Fig). Lysing matrix E was used in the present study because of the Cq values on the qPCR seemed to be less disperse compared to the Cq values obtained with matrix H. Although, no differences on the Cq values were observed when clot samples, from individuals known to be positive by qPCR in GEB (n = 10, *P = 0*.*5340*,S1C Fig) or when Cq values for the IAC were compared on samples extracted with lysing matrix E or H (n = 28; *P = 0*.*5310*, S1D Fig).

Because the normalization value in all plates was close to 1.0 (mean correction factor for GEB = 1.00136 and mean correction factor for CLOT = 0.99737), the original Cq values were used in the final analysis.

**Fig 1:**
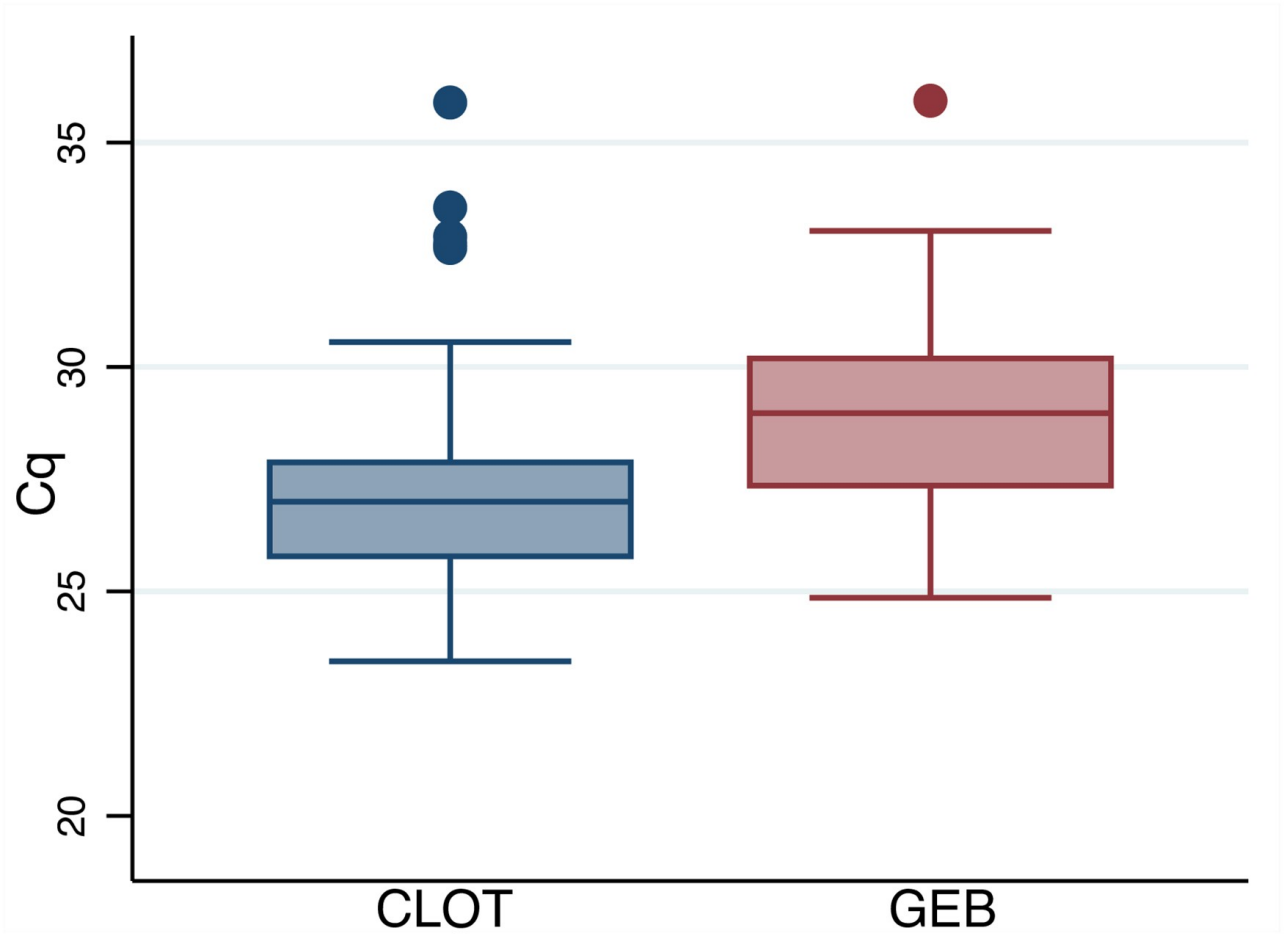
Distribution of Cq values in GEB and clot samples. The line inside the box represents the median, and the box comprises the lower and upper quartiles. The mean Cq for clot samples (extracted using the new technique) was 27.32 [95%CI: 26.69–27.95]. The mean Cq for GEB samples was 29.02 [95% CI: 28.36–29.67] (*P = 0003*). Agreement between the two samples was 95.9% (Kappa = 0.8483). Out of the five samples that tested positive in the clot extracted DNA with a Cq value greater than 32, three were also positive on the qPCR using DNA from GEB samples.

Out of the 265 samples analyzed, 150 samples were from Chagas seropositive individuals. The mean DNA concentration was higher on clot samples than in GEB samples (S1 Table). Among the Chagas seropositive samples, the qPCR tested positive in 31.3% (47/150) [95% CI: 24.02–39.41] of GEB samples (Table 1) and in 40.0% (60/150) [95% CI: 32.09–48.31] of clot samples (Table 2) extracted using the new technique. When the proportion of qPCR positive samples using, DNA extracted from clot samples were compared to the proportion of positives using DNA extracted from GEB using the McNemar’s test (Table 3), the difference in the proportions was statistically significant (*P = 0*.*0002*). Similarly, when the mean Cq values obtained by the qPCR were compared, the mean Cq value was significantly lower, when qPCR was performed using DNA extracted from clot samples, than using DNA extracted from GEB samples 27.32 [CI: 26.69–27.95] and 29.02 [CI: 28.36–29.67], respectively (*P* < 0.0003) (Fig 1). The agreement between the qPCR using clot DNA and GEB DNA was 95.9% (*Kappa = 0*.*8483*, CI:0.769–0.928).

**Table 1:** Comparison of qPCR to serology using DNA extracted from GEB.

		SEROLOGY		Total
		Positive	Negative	
GEB	Positive	47	0	47
	Negative	103	115	218
	Sensitivity (95% CI)	31.3% (24.0–39.4%)		
	Specificity (95% CI)	100% (96.8–100%)		
Total		150	115	265

Sensitivity and specificity of the qPCR using GEB samples was calculated considering the serology (positive to all ELISA, HAI and Western blot positive) as the gold standard.

**Table 2:** Comparison of qPCR to serology using DNA extracted from clot.

		SEROLOGY		Total
		Positive	Negative	
CLOT	Positive	60	0	60
	Negative	90	115	205
	Sensitivity (95% CI)	40.0% (32.1–48.3%)		
	Specificity (95% CI)	100% (96.8–100%)		
Total		150	115	265

Sensitivity and specificity of the qPCR using clot samples was calculated considering the serology (positive to all ELISA, HAI and Western blot positive) as the gold standard.

**Table 3:** Comparison of qPCR using DNA extracted from GEB or clot.

		GEB-qPCR		Total
		Positive	Negative	
**CLOT–qPCR**	Positive	47	13	60
	Negative	0	205	205
Total		47	218	265
*Kappa* (95% CI)		*0*.*8483* (0.769–0.928)		

The proportion of samples identified as positive when DNA extracted from clot was higher than when DNA extracted from GEB was used (McNemar’s *P = 0*.*0002*).

## Discussion

Chagas disease is still considered an important health problem globally. The current diagnosis is based on antibody detection by several methods, however the presence of antibody may not necessarily represent current infection. *Trypanosoma cruzi* DNA detection has been increasingly used not only as a diagnostic technique but also as a surrogate for treatment failure [11,26] We have previously shown that for PCR based diagnosis of Chagas, DNA extraction of clot samples shows better sensitivity than either whole blot or buffy coat [14]. However, phenol-chloroform based DNA extraction has several drawbacks, including toxicity and carcinogenicity of phenol/chloroform. Because of the complicated DNA extraction procedure, samples are more prone to cross-contamination. Here we have developed and improved technique for DNA extraction from clot samples. The technique is based on the initial disruption of clot using the fast prep machine followed by the internationally standardized DNA extraction protocol for the molecular diagnosis of Chagas disease for GEB samples.

The qPCR using DNA extracted from GEB samples has been standardized to be used as a universal diagnostic technique [12,14,20,27,28]. Unfortunately, shipping and handling of Guanidine and GEB samples has become troublesome because of the International Air Transport Association (IATA) regulations. Moreover, PCR performed using DNA extracted from clot shows higher sensitivity than PCR using DNA extracted from whole blood [14]. Although, two samples tested positive in qPCR from clot (Cq 32.74 and 35.89) and negative in the qPCR (Cq >40) using DNA from GEB, a one to one dilution of the whole blood with the GE only explains a Cq difference of 1.66, considering an efficiency of 100%.

It should be noted that serology in chronic cases of Chagas infection is more sensitive than qPCR. In contrast, in acute infection with congenital infection qPCR is more sensitive than serology [1]. Thus, serology might not be an adequate gold standard, comparing sensitivity of the qPCR to serology might not be the best approach.

Although the use of GE for DNA preservation and sample shipping is avoided; the technique described here still depends on the use of GE for DNA extraction. Guanidine is a chaotropic agent that helps to disrupt the clot and the cell membranes, and solubilizes the DNA. Other home-made and commercial buffers to disrupt the clot have been tested by us with results that were not comparable to the ones obtained using GE.

By using DNA extracted from clot, the sensitivity of the qPCR is increased as reflected by the low Cq values obtained in comparison to the Cq values obtained using DNA extracted from GEB. Several DNA extraction methods from clot samples have been reported previously, most of them oriented to the recovery of DNA from human (host) origin[29–32] or from intracellular microorganisms [33]. *T*. *cruzi*, if present in the blood, is not intracellularly located. Free DNA from parasite origin seems to be circulating in the bloodstream, as DNA has been successfully recovered from serum samples by PCR detection [34], moreover injected *T*. *cruzi* kDNA in mice circulates in blood for at least 48 hrs [35]. Clot probably traps the parasites and the DNA present in the blood, thus concentrating them. In addition, washing the clot with water or with erythrocyte lysing buffers as previously reported [14,33] most likely lyses the *T*. *cruzi* parasites releasing the genetic material, which will also be washed out along with hemoglobin. Thus, washing the clot might lead to decrease the sensitivity of any technique targeting the *T*. *cruzi* DNA, such as the qPCR. Our protocol avoided any washing steps of the clot previous to the DNA purification to ensure that most of the DNA from the parasites could be recovered from the sample. Additionally, the use of individually packed FastPrep Lysing matrix ensures that cross contamination is almost completely eliminated during sample extraction.

Improving the sensitivity of the techniques that demonstrate the presence of the parasite during Chagas disease constitutes an important advancement in the diagnosis of Chagas disease. Contrary to the detection of antibodies, which is an indicator of either active chronic infection or merely exposure to the parasite, detection of parasite DNA is generally an indication of active infection, as detection of *T*. *cruzi* genetic material is consider equivalent to parasite detection[36]. Improvement of qPCR sensitivity is especially important for identifying active infection in those individuals with very low blood parasite loads, such as those with chronic or indeterminate phase where it is used for assaying response to treatment. However, our results shown a low sensitivity of the qPCR compared to serology suggesting that the qPCR technique might still need further improvement.

In summary, we describe here an improved methodology for the detection of *T*. *cruzi* DNA using clot samples which might improve early diagnosis and therefore will improve treatment and prognosis of infected individuals, especially of those with low blood loads of parasites.

## Supporting information

S1 Fig Evaluation of different lysing matrix for DNA extraction from clot samples. Initial evaluation of different lysing was performed using clot samples spiked with 5 x 10^6^ parasites/ml (A) and 1 x 10^6^ parasites/ml (B). Additionally, lysing matrix E and H were further evaluated using clot samples from individuals known to be positive by qPCR using GEB samples (C). Cq values for the IAC on negative samples were also analyzed (D). The use of lysing matrix C and J were discarded because of destruction of the lysing matrix components.
(DOCX)

S1 Table DNA yield of samples extracted from clot or GEB samples. DNA concentration was measured by spectrophotometry using the NanoDrop 2000 Spectrophotometer (Thermo Scientific, USA).
(DOCX)

S2 Table Cq values for the internal amplification control from DNA extracted from clot or GEB samples. (DOCX)
