# Supplementary material for: Improved DNA extraction technique from clot for the diagnosis of Chagas disease
Source: PLoS Negl Trop Dis. 2019 Jan 11;13(1):e0007024. doi: 10.1371/journal.pntd.0007024 (PMC6329489; doi:10.1371/journal.pntd.0007024)
Supplement: S1 Fig — Initial evaluation of different lysing was performed using clot samples spiked with 5 x 10^6^ parasites/ml (A) and 1 x 10^6^ parasites/ml (B). Additionally, lysing matrix E and H were further evaluated using clot samples from individuals known to be positive by qPCR using GEB samples (C). Cq values for the IAC on negative samples were also analyzed (D). The use of lysing matrix C and J were discarded because of destruction of the lysing matrix components. (DOCX) [file pntd.0007024.s001.docx]

**S1 Fig. Evaluation of different Lysing Matrix for DNA extraction from clot samples**


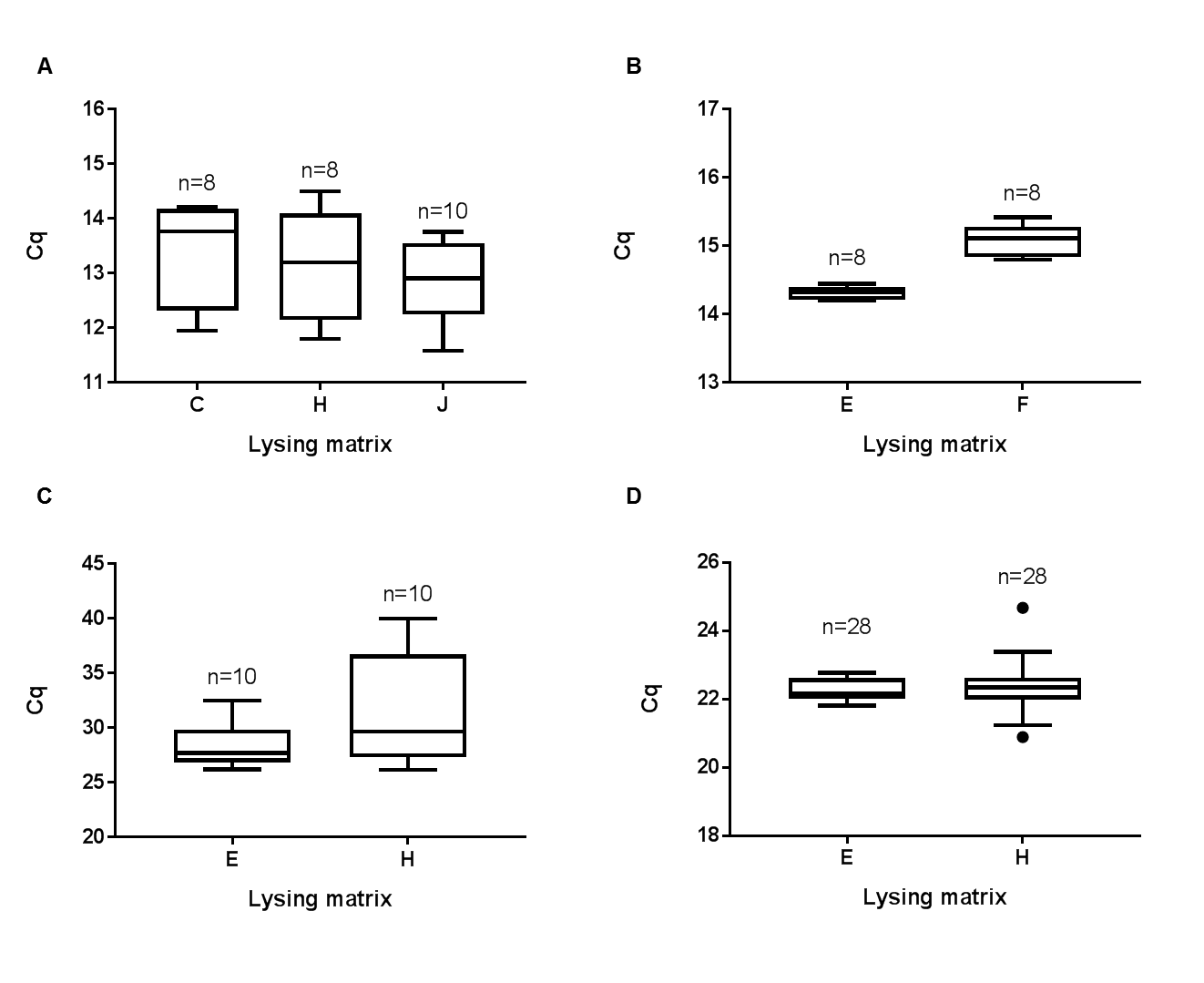


Initial evaluation of different lysing was performed using clot samples spiked with 5 x 10^6^ parasites/ml (A) and 1 x 10^6^ parasites/ml (B). Additionally, lysing matrix E and H were further evaluated using clot samples from individuals known to be positive by qPCR using GEB samples (C). Cq values for the IAC on negative samples were also analyzed (D). The use of lysing matrix C and J were discarded because of destruction of the lysing matrix components.
