# Supplementary material for: Improved DNA extraction technique from clot for the diagnosis of Chagas disease
Source: PLoS Negl Trop Dis. 2019 Jan 11;13(1):e0007024. doi: 10.1371/journal.pntd.0007024 (PMC6329489; doi:10.1371/journal.pntd.0007024)
Supplement: S1 Table — DNA concentration was measured by spectrophotometry using the NanoDrop 2000 Spectrophotometer (Thermo Scientific, USA). (DOCX) [file pntd.0007024.s002.docx]

**S1 Table. DNA yield of samples extracted from clot or GEB samples.**

| Sample |  | Variables |  | Mean |  | 95% CI |
| --- | --- | --- | --- | --- | --- | --- |
| CLOT |  | DNA Concentration (ng/µl) |  | 144.4876 |  | 133.8535 – 155.1216 |
|  |  | 260/280 Ratio |  | 1.8437 |  | 1.8382 – 1.8492 |
|  |  | 260/230 Ratio |  | 2.1029 |  | 2.0699 – 2.1358 |
| GEB |  | DNA Concentration (ng/µl) |  | 64.3368 |  | 57.5730 – 71.1005 |
|  |  | 260/280 Ratio |  | 1.8070 |  | 1.7915 – 1.8225 |
|  |  | 260/230 Ratio |  | 1.6696 |  | 1.5863 – 1.7528 |

DNA concentration was measured by spectrophotometry using the NanoDrop 2000 Spectrophotometer (Thermo Scientific, USA).
