# Supplementary material for: Improved DNA extraction technique from clot for the diagnosis of Chagas disease
Source: PLoS Negl Trop Dis. 2019 Jan 11;13(1):e0007024. doi: 10.1371/journal.pntd.0007024 (PMC6329489; doi:10.1371/journal.pntd.0007024)
Supplement: S2 Table — (DOCX) [file pntd.0007024.s003.docx]

**S2 Table. Cq values for the Internal Amplification control from DNA extracted from clot or GEB samples.**

| Sample |  | Cq Values for the Internal Control Amplification | | | | | | |
| --- | --- | --- | --- | --- | --- | --- | --- | --- |
|  |  | Median |  | 25^th^-75^th^ percentile |  | Mean |  | 95% CI |
| CLOT |  | 21.29 |  | 20.34 – 22.04 |  | 21.14 |  | 21.01 – 21.27 |
| GEB |  | 20.82 |  | 20.37 – 21.56 |  | 20.89 |  | 20.77 – 21.01 |

Because extraction was performed independently, and the batch of IAC was not the same along all the study the Cq values might not necessarily reflect a difference in the extraction method.
